# CANCER SURVIVORSHIP AND SUBJECTIVE COGNITIVE DECLINE IN THE US: RESULTS FROM A NATIONALLY REPRESENTATIVE SAMPLE

**DOI:** 10.1093/geroni/igad104.0091

**Published:** 2023-12-21

**Authors:** Monique Brown, Ally Hucek, Jingkai Wei, Loretta Anderson, Muzi Na

**Affiliations:** University of South Carolina, Columbia, South Carolina, United States; University of South Carolina, Columbia, South Carolina, United States; University of South Carolina, Columbia, South Carolina, United States; University of Maryland Baltimore, Baltimore, Maryland, United States; Penn State, University Park, Pennsylvania, United States

## Abstract

Cancer may be associated with objective cognitive decline. However, studies examining the link between cancer and subjective cognitive decline (SCD), which occurs earlier during cognitive trajectories, are lacking. In addition, research assessing risk factors for SCD among cancer survivors is scant. Therefore, this study aimed to examine the association between cancer and SCD in the past year, and assess the association between age of diagnosis, cancer treatment, and SCD among cancer survivors. Data were obtained from the 2021 Behavioral Risk Factor Surveillance System (N=15,447). Crude and adjusted logistic regression models, controlling for sociodemographic characteristics, were used to determine the association between cancer diagnosis, age at diagnosis, cancer treatment, and SCD. The association between ever having cancer and SCD in the past year was not statistically significant. However, disparities occurred among those who were cancer survivors. For example, those who were diagnosed with cancer in middle age (aged 35 to 44) had higher odds of SCD compared to being diagnosed at aged 45 and older (adjusted OR (aOR): 2.25; 95% CI: 1.10 – 4.58). Those who were currently undergoing treatment were more likely to have SCD compared to those who completed treatment (aOR: 1.86; 95% CI: 1.06 – 3.27). Respondents who had not started treatment and those who reported treatment was not necessary also had higher odds of SCD compared to those who completed treatment. Findings suggest that age and treatment disparities exist in SCD among cancer survivors. Future studies may include longitudinal observational studies examining cancer-related risks and SCD.

